# Tie-2, G-CSF, and Leptin as Promising Diagnostic Biomarkers for Endometrial Cancer: A Pilot Study

**DOI:** 10.3390/jcm10040765

**Published:** 2021-02-15

**Authors:** Luka Roškar, Teja Klančič, Tamara Knific, Tea Lanišnik Rižner, Špela Smrkolj

**Affiliations:** 1 Department of Obstetrics and Gynaecology, Faculty of Medicine, University of Ljubljana, 1000 Ljubljana, Slovenia; luka.roskar@outlook.com (L.R.); spela.smrkolj@mf.uni-lj.si (Š.S.); 2 Institute of Biochemistry, Faculty of Medicine, University of Ljubljana, 1000 Ljubljana, Slovenia; teja.klancic@mf.uni-lj.si (T.K.); tamara.knific@mf.uni-lj.si (T.K.); 3 Department of Obstetrics and Gynaecology, University Medical Centre, 1000 Ljubljana, Slovenia

**Keywords:** biomarkers, angiogenesis, endometrial cancer, angiogenic factors, Tie-2, G-CSF, leptin

## Abstract

Preoperative determination of the extent of endometrial cancer (EC) would avoid the complications associated with radical surgery. Screening of patients’ plasma biomarkers might enable a more precise diagnosis of EC and a tailored treatment approach. This prospective case-control monocentric pilot study included 76 postmenopausal women (38 endometrioid EC patients and 38 control patients with benign gynecological conditions), and 37 angiogenic factors (AFs) were investigated as potential biomarkers for EC. AF concentrations in preoperative plasma samples were measured using Luminex xMAP™ multiplexing technology. The plasma levels of sTie-2 and G-CSF were significantly lower in EC compared to control patients, whereas the plasma levels of leptin were significantly higher in EC patients. Neuropilin-1 plasma levels were significantly higher in patients with type 2 EC (grade 3) compared to patients with lower grade cancer or controls. Follistatin levels were significantly higher in patients with lymphovascular invasion, and IL-8 plasma levels were significantly higher in patients with metastases. If validated, the plasma concentrations of the indicated AFs could represent an important additional diagnostic tool for the early detection and characterization of EC. This could guide the decision-making on the extent of surgery. Further studies with larger patient numbers are currently ongoing.

## 1. Introduction

Endometrial cancer (EC) is a leading gynecological malignancy in the developed world and its incidence is rapidly increasing [1,2]. Classification according to endocrine and metabolic disturbances divides endometrial cancer into two types. Well- or moderately differentiated endometrioid EC represents prognostically favorable type 1 EC, whereas poorly differentiated endometrioid EC represents prognostically less favorable type 2 EC with a tendency for deep myometrial invasion (DMI) and metastasis [3,4,5]. The average 5-year overall survival rates are 85.6% and 58.8% for type 1 and 2 EC, respectively [4].

While this classification into two types is still widely in use, a newer molecular classification according to genomic, transcriptomic, and proteomic characterizations was proposed in 2014, dividing EC into four categories and offering a more tailored therapeutic approach [6]. Recently it has been demonstrated that the integration of molecular profiling into a daily routine is possible and is expected to change treatment decisions [7]. Nevertheless, the diagnosis and treatment strategy for EC are still mostly defined according to the histological findings of endometrial biopsies [8]. The most important histological findings, which determine the extension of further surgical therapy, are the type and grade of EC as well as the presence of DMI or lymphovascular invasion (LVI) [9,10]. In terms of histopathology, endometrioid EC is categorized as being well differentiated (G1), moderately differentiated (G2), and poorly differentiated (G3) [11].

Frequently, the histological findings from biopsies do not correlate with the histological findings after hysterectomy, and thus additional methods are needed to more precisely determine the extent of the disease [12,13,14]. Plasma biomarkers represent very important diagnostic, therapeutic, and prognostic tools for the treatment of different malignancies. Despite the frequency of EC versus other malignancies, there is currently still no biochemical screening with diagnostic or prognostic markers, other than CA-125 and HE4, available in clinical practice [9,15]. The discovery of new plasma biomarkers would enable a more precise diagnosis of EC and a tailored treatment approach [16].

**Figure 1:**
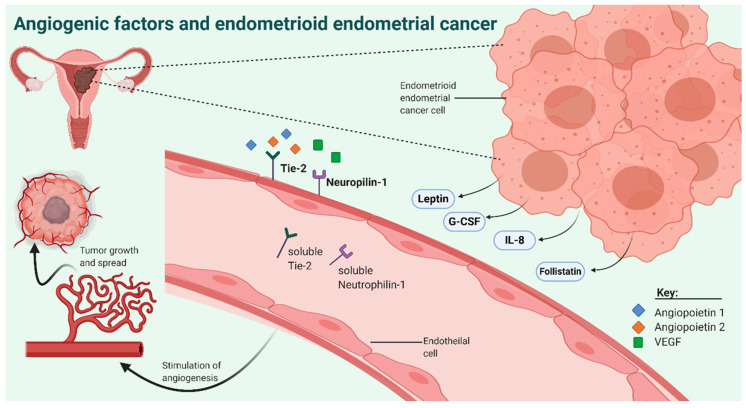
The angiogenic factors involved in the pathology of endometrioid EC. Created with BioRender.com.

One group of potential biomarkers are angiogenic factors (AFs) that comprise relatively small molecules, usually cytokines or chemokines, the production of which is influenced by cancerous tissue [17,18]. AFs include both proangiogenic factors like VEGF, and inhibitors of angiogenesis like angiostatin. In cancer cells, production of AF is dysregulated to enable the faster sprouting of new vessels (Figure 1) [19]. The main trigger that influences AF production is the lack of oxygen that occurs due to insufficient diffusion when cancerous tissue is located 0.1–0.2 mm from the blood vessels [18,20]. At this stage, AFs transform the surrounding vascular tissue into a supply network that enables the fast growth, spread, and metastasis of cancer [21]. The production of AFs by cancer cells leads to altered AF levels in the surrounding tissue and blood plasma [22,23]. Altered AF levels may thus represent potential markers, which could detect cancer from blood plasma samples in the early and prognostically favorable stages of cancer [24]. The plasma concentrations of AFs could also represent an important additional diagnostic tool for a more precise diagnosis of EC; this could guide decision-making regarding the extent of surgery. This could be limited to hysterectomy and bilateral adnexectomy or could even be extended to the more radical procedure in which also pelvic and para-aortic lymphadenectomy is performed. These extended procedures have a higher rate of possible complications and postoperative morbidity, such as permanent lymphedema, ascites, or nerve damage [15,25,26].

In the present study, we evaluated the concentrations of 37 different AFs in preoperative plasma samples of patients with endometrioid EC and control patients with benign gynecological conditions (e.g., prolapsed uterus or chronic pelvic pain).

## 2. Experimental Section

### 2.1. Patient Enrolment

Patient enrolment took place at the Department of Obstetrics and Gynecology, University Medical Centre Ljubljana, Slovenia. In this monocentric case-control study, we included 76 women who underwent surgical treatment, including a group of EC patients (n = 38) and a control group of women with prolapsed uteri or myoma (n = 38). Women were excluded from the study if they were of reproductive age or had other non-endometrioid EC malignancies, HIV infection, or acute inflammation. This study was approved by the National Medical Ethics Committee of the Republic of Slovenia (No. 0120-515/2017/4), and all participants signed written informed consent before participating in this study.

The patients were recruited by senior gynecologists with the help of study nurses. One day to one week prior to surgery, morning blood samples were collected, and additional information was obtained regarding lifestyle, medication used, and gynecological and clinical status (Table 1 and Supplementary Tables S1 and S2). For sample collection and processing, strict and detailed standard operating procedures were followed, and plasma samples were stored at −80 °C until further analysis.

### 2.2. Measurements of AFs

All plasma samples were tested for 37 circulating angiogenesis biomarkers using Luminex xMAP multiplexing technology with two Milliplex^®^ MAP Human Angiogenesis/Growth Factor Magnetic Bead Panels: 20-plex HANG2MAG-12K and 17-plex HAGP1MAG-12K (Merck Millipore, Burlington, Massachusetts, USA, LOT#HAGP2-8012-2 and LOT#3282193, respectively). All tests were performed according to the manufacturer’s protocol. Limit of detection (LOD) for 37 angiogenesis biomarkers are included in Supplementary Table S3. Briefly, the method is based on 5.5 μm polystyrene beads that are labelled with two different fluorescent dyes at different ratios assigned for each individual antibody, thus enabling the quantification of 37 different angiogenesis biomarkers (Table 2). The samples were anonymized, and the person performing the assays was blind to the identity of the samples. xPonent 4.2 Software, Luminex, Austin, Texas, USA, with five-parameter logistic regression modelling was used to calculate the final concentrations.

The same plasma samples were previously analyzed using electrochemiluminescent immunoassays specific for CA-125 and HE4 on a Cobas e411 immunoassay analyzer (Roche Diagnostics GmbH, Manheim, Germany). Quantitative detection kits for CA-125 (REF: 11776223190, LOT: 139788-01) and HE4 (REF: 05950929190, LOT: 112732-01) were used [27].

### 2.3. Statistics

The differences in plasma AF levels between EC patients and control patients were analyzed, as were the differences between EC patients with or without lymphovascular invasion and deep myometrial invasion, EC patients with different grades of the disease, and EC patients with or without metastasis.

The Shapiro–Wilk test was used to determine the normality of the distributions and as the data were not normally distributed the two-sided Mann–Whitney U test was used for univariate statistical analysis of differences between EC patients and control patients. The non-parametric Kruskal–Wallis test with Dunn’s multiple comparison corrections as post-hoc tests was used to compare more than two groups. The chi-square test was used to compare categorical variables. The results of the descriptive analysis (i.e., the patient’s clinical data) were presented as mean ± standard deviation, while the concentrations of the measured proteins were presented as median and range (Table 2). Statistical significance was set at *p* < 0.05. Receiver operating characteristic (ROC) curves for the angiogenic biomarkers CA-125 and HE4 were used to compare the separation between EC and control patients.

## 3. Results

### 3.1. Characteristics of the EC Patients and Control Patients

The case group comprised 38 EC patients with a mean age of 65.9 ± 8.2 years (range: 52–88 years) and mean body mass index (BMI) of 31.8 ± 6.1 kg/m^2^ (range: 20.6–45.7 kg/m^2^). The detailed clinical characteristics are presented in Table 1 and in Supplementary Table S1.

LVI was observed in 5 patients (13.2%), DMI in 12 patients (31.6%), <50% invasion into the myometrium in 15 patients (39.5%), and no invasion into the myometrium in 11 patients (28.9%). According to the classification of the International Federation of Gynecology and Obstetrics [28], the following EC stages were observed: IA (n = 23, 60.5%), IB (n = 9, 23.7%), IIIA (n = 2, 5.3%), IIIB (n = 1, 2.6%), and IIIC (n = 3, 7.9%).

The control group included 38 patients with a mean age of 66.9 ± 8.4 years (range: 52–83 years) and mean BMI of 27.5 ± 3.9 kg/m^2^ (range: 21.6–38.7 kg/m^2^). The detailed clinical characteristics are presented in Table 1 and Supplementary Table S1.

When both groups were compared, there was a statistically significant difference in BMI (*p* = 0.001); however, there were no differences in age distribution, hormonal therapy, medication intake, or smoking status (Table 1 and Supplementary Tables S1 and S2). None of the included patients received drugs with known anti-angiogenic effect and no neoadjuvant chemotherapy was used.

### 3.2. Levels of AFs in EC and Control Patients

**Figure 2:**
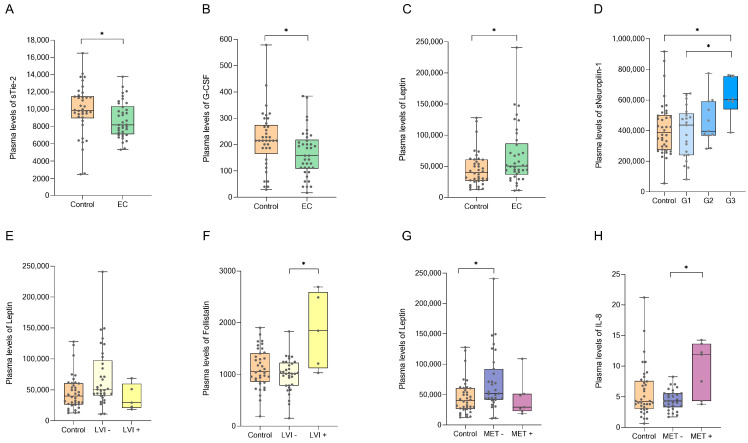
Box plots (min to max with all points shown) comparing plasma levels of angiogenic biomarkers (pg/mL) between study groups. (**A**–**C**) Control and endometrial cancer (EC) patients. (**D**) Control patients and patients with different grades of EC. (**E**,**F**) Control patients and patients with or without lymphovascular invasion (LVI). (**G**,**H**) Control patients and patients with or without metastasis (MET). * signifies *p* < 0.05.

All 37 AFs were measured in all plasma samples. Univariate statistical analysis revealed statistically significant differences in the plasma concentrations of three AFs between EC and control patients (Table 2, Figure 2). The plasma levels of sTie-2 and G-CSF were significantly decreased in EC patients compared to those of control patients (*p* < 0.05). Within the EC group, Tie-2 levels were lower in patients with LVI and DMI; however, these differences did not reach statistical significance. Additionally, G-CSF levels were insignificantly lower in patients with DMI.

Leptin levels were significantly elevated in EC versus control patients, and the difference was most distinguished, although not significant, in patients with poorly differentiated, grade 3 tumors. Similarly, neuropilin-1 levels were significantly higher in patients with grade 3 cancer than in patients with lower grade cancer or controls (*p* < 0.05).

Leptin levels were insignificantly elevated in patients with locally limited disease without LVI, yet decreased in patients with disseminated disease with LVI. This is probably due to the higher BMI of patients without LVI (the mean BMI values were 32.1 kg/m^2^ and 29.9 kg/m^2^ in patients without and with LVI, respectively), as leptin is known to be increased in more obese patients [29]. Interestingly, follistatin levels were significantly higher in patients with LVI, and IL-8 levels were significantly higher in patients with metastasis (Figure 2). However, these findings should be considered with caution and confirmed in a larger group of patients, as our cohort only included five patients with LVI and seven patients with metastases.

**Figure 3:**
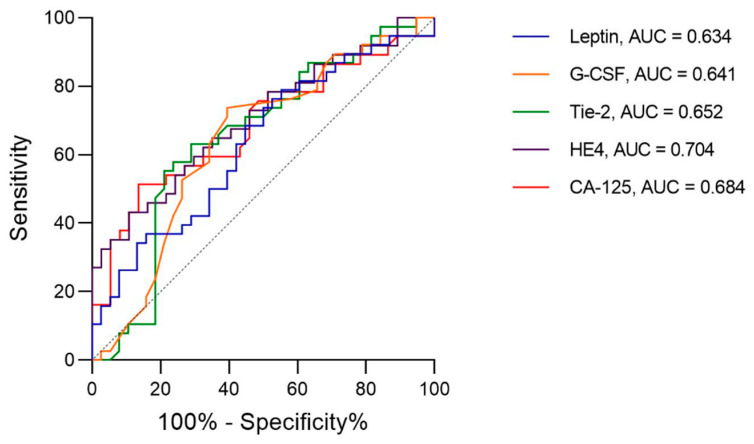
Diagnostic receiver operating characteristic (ROC) curves with area under the curve (AUC) values for three angiogenic biomarkers compared to CA-125 and HE-3.

The diagnostic characteristics of three AFs with detected differences in plasma levels between EC and control patients were compared to those of CA-125 and HE4, previously measured in our lab [27]. ROC curve analysis revealed a slightly lower area under the curve (AUC) values for three AFs compared to those of CA-125 and HE4. The highest AUC value of 0.65 was determined for Tie-2, followed by 0.64 and 0.63 for G-CSF and leptin, respectively (Figure 3).

## 4. Discussion

Increased levels of pro-angiogenic factors were previously demonstrated in EC tissue by immunohistochemistry [30]. However, a clinically less invasive and faster approach to detect increased angiogenesis is to examine plasma levels of AFs. Unlike immunohistochemical analyses, analyses of plasma AFs in EC patients have rarely been performed [3]. Our study has demonstrated differences in the plasma levels of Tie-2, G-CSF, and leptin between EC and control patients. Appropriate validation and studies on larger groups of patients may reveal that these proteins have the potential to be included into diagnostic models, which could lead to earlier and more tailored EC therapy in the future.

Our study also showed increased plasma levels of IL-8 in metastatic cases of endometrioid EC, which is in accordance with a previous in vitro study on cell lines [31]. Currently, EC staging is mainly assessed by histological examination of pelvic and para-aortic nodules that are obtained by lymphadenectomy [9]. Therefore, plasma levels of IL-8 in EC patients should be further evaluated as a staging tool that could present a less invasive alternative to the current lymphadenectomy.

The cytokine VEGF has been proven as an important AF in different types of cancer [32,33]. Its ability to increase vessel permeability and endothelial proliferation as well as its antiapoptotic effect make it the most researched AF and potential target for anticancer targeted therapy [34,35]. However, due to cyclic proliferation, endometrial tissue undergoes a repetitive physiological process of angiogenesis every month. Studies have demonstrated different tissue levels of VEGF between the proliferative and secretory phase [36,37]. To exclude the impact of cyclic steroid hormone levels on angiogenesis, our study included only postmenopausal women.

Previous immunohistochemical studies are inconclusive about the importance of VEGF in EC tissue. While some animal models also showed the importance of VEGF in EC [19,38], other studies did not observe higher VEGF levels in EC tissue [36,39]. However, Shaarawy and El-Sharkawy reported higher VEGF levels in serum from patients with endometrial hyperplasia and endometrial cancer in the comparison to the control group of postmenopausal women. They also observed significantly decreased VEGF levels after treatment [40]. In our study, we did not detect differences in VEGF plasma levels between EC and control patients. Nonetheless, the levels of neuropilin-1, an essential co-receptor for VEGF, were increased in poorly differentiated (G3) EC patients. This is in accordance with the immunohistochemical study by Okon et al. [41], which demonstrated that atypical neuropilin-1 expression in endometrial tissue may serve as a biomarker for metastatic endometrial tumors. Another study demonstrated that neuropilin-1 was exclusively present in cancer cells, as opposed to the control samples, and at distinctly higher levels in G2 and G3 than those in G1 [42].

Our results indicate that follistatin levels are elevated in EC patients with LVI. To the best of our knowledge, follistatin has not been previously analyzed in blood samples from EC patients. However, one study demonstrated higher serum follistatin levels in ovarian cancer patients compared to patients with benign ovarian cysts [43]. At the mRNA level there were no significant differences in expression of follistatin when comparing atrophic endometrial tissue to adenocarcinoma tissue [44]. However, The Cancer Genome Atlas (TCGA) data indicate that EC patients with higher follistatin mRNA levels have a decreased 5-year survival, which implies that follistatin has important role in the progression of endometrial cancer (https://www.proteinatlas.org) [45].

Angiopoietins are, after VEGF, the second most studied cytokine family with angiogenic properties [46]. Their receptor Tie-2 is essential in the remodeling and maturation of blood and lymphatic vessels. As a receptor for both angiopoietin-1 and -2, it has both pro- and anti-angiogenic properties [47,48]. One immunohistochemical study showed that Tie2 expression in endothelial cells tended to be lower in G2 and G3 endometrial adenocarcinoma than in normal endometrium [36]. Correspondingly, our study has revealed significantly lower Tie-2 plasma levels in EC patients.

Leptin is well known for its role in the regulation of energy homeostasis, neuroendocrine function, and metabolism [49]. Furthermore, it also induces endothelial cell proliferation and the expression of metalloproteinases [50]. In our study, EC patients had significantly higher leptin plasma levels; however, they also had significantly higher BMI values.

There are only a few clinical case reports of tumor G-CSF production in gynecologic malignancies. The elevated plasma values were mainly reported in highly advanced non-endometrioid EC with poor prognosis or a large tumor burden [51,52,53]. In our study, EC patients had lower G-CSF plasma levels; however, the median values of both cancer and control groups remained relatively low compared to the case reports with advanced type 2 EC, evaluated via the commercially available ELISA kit [54].

To compare the diagnostic value of the studied AFs with biomarkers that are in clinical use, we compared the ROC curves for sTie-2, G-CSF, and leptin with those for CA-125 and HE4. CA-125 and HE4 have been extensively studied in ovarian and EC, and their diagnostic and prognostic values in gynecological cancers have been reported [16,55,56,57]. This comparison revealed that sTie-2, G-CSF, and leptin have similar AUC values as CA-125 and HE4 for the separation of EC and control patients.

To conclude, the results of our study indicate that the plasma levels of different AFs might be involved in the growth of endometrioid EC. The plasma levels of G-CSF, Tie-2, and leptin significantly differ between EC and control patients. The plasma concentrations of these AFs could represent an important additional diagnostic tool for the early detection and characterization of EC and could guide the decision-making regarding the extent of surgery. Further validation studies with larger patient numbers are currently ongoing.

## Figures and Tables

**Table 1:** Detailed clinical characteristics of the study participants.

		Control Patients n = 38 (100%)	EC Patients n = 38 (100%)	*p*^a^ Values
Age category	50–59.9 years	10 (26.3)	11 (28.9)	ns
	60–69.9 years	12 (31.6)	14 (36.8)	
	70–79.9 years	15 (39.5)	12 (31.6)	
	>80 years	1 (2.6)	1 (2.6)	
Body mass index (kg/m^2^)	18.6–24.9 (normal weight)	10 (26.3)	4 (10.5)	0.001
	25–29.9 (overweight)	16 (42.1)	15 (39.5)	
	30–34.9 (class I obesity)	6 (15.8)	9 (23.7)	
	35–39.9 (class II obesity)	1 (2.6)	6 (15.8)	
	40–49.9 (class III obesity)	0 (0)	4 (10.5)	
	Missing data	5 (13.2)	0 (0)	
Smoking status	Nonsmoker	29 (76.3)	30 (78.9)	ns
	Smoker	2 (5.3)	3 (7.9)	
	Occasional smoker	2 (5.3)	2 (5.3)	
	Former smoker	3 (7.9)	3 (7.9)	
	Missing data	2 (5.3)	0 (0)	
Hormonal therapy in the past	No	21 (55.3)	25 (65.8)	ns
	Yes	3 (7.9)	1 (2.6)	
	Missing	14 (36.8)	12 (31.6)	
Peroral contraception in the past	No	18 (47.4)	16 (42.1)	ns
	Yes	7 (18.4)	7 (18.4)	
	Missing	13 (34.2)	15 (39.5)	
Medication intake in last 7 days	No	0 (0)	1 (2.6)	ns
	Yes	33 (86.8)	28 (73.7)	
	Missing data	5 (13.2)	9 (23.7)	

^a^*p* values were calculated using the non-parametric Mann–Whitney test for continuous variables and the chi-squared test for categorical variables; ns = not significant.

**Table 2:** Plasma concentrations (pg/mL) of the measured angiogenesis biomarkers.

	Control Patients n = 38		EC Patients n = 38		*p*^a^ Values (<0.05)
	Median	Range	Median	Range	
angiostatin	68,762.7	26,019.6–131,674.6	68,968.4	10,337.8–133,835.1	
sAXL	1352.0	447.3–2214.6	1400.2	487.3–1932.0	
sc-Kit/SCFR	25,473.2	6497.9–44,838.5	27,755.8	6382.0–47,182.2	
sHer2	4686.2	2750.8–6958.6	4589.5	2247.6–7616.4	
sHer3	5310.9	735.3–7160.7	4921.2	2174.1–8658.4	
sE-Selectin	88,127.2	42,351.2–164,728.6	84,245.3	35,178.1–132,759.8	
sHGFR/c-Met	39,388.4	12,640.9–86,504.3	38,925.6	22,210.5–74,766.1	
Tenascin C	11,069.2	1242.1–22,237.3	10,313.6	1179.9–18,276.6	
PDGF-AB/BB	921.7	266.2–2355.9	914.4	338.5–9049.4	
sIL-6Ralpha	26,213.5	8505.9–40,310.4	27,467.1	5581.6–42,348.2	
sTie-2	9862.9	2465.8–16,502.5	8191.4	5314.6–13,762.4	0.0218
Thrombospondin-2	8696.5	1347.0–26,885.0	7591.7	910.6–18,021.5	
sNeuropilin-1	387,531.5	55,935.1–916,924.7	459,548.5	81,804.2–775,122.2	
sEGFR	1157.7	321.9–1949.8	1212.5	386.9–2136.5	
suPAR	9886.1	3055.3–18,468.5	10,253.5	4365.7–16,059.8	
sVEGFR1	1049.8	114.8–2381.0	979.1	142.4–2116.0	
sVEGFR3	14,534.9	1908.7–28,017.4	12,534.2	546.7–26,452.6	
sPECAM-1	5301.3	1907.4–7014.2	4814.0	1364.5–7573.7	
Osteopontin	4620.1	1745.7–9509.6	4037.6	1721.5–9583.0	
sVEGFR2	12,044.6	6781.0–17,613.6	12,520.4	6115.8–18,684.0	
Angiopoietin-2	2442.0	594.3–7937.8	2209.4	733.3–5191.5	
BMP-9	103.2	7.9–244.7	110.1	19.2–847.6	
Endoglin	1741.2	589.4–3370.5	1730.9	364.4–2808.5	
Follistatin	1054.8	190.3–1901.1	1032.3	153.4–2692.5	
G-CSF	214.8 ^c^	29.0–578.6	158.1 ^c^	16.8–384.3	0.0175
HB-EGF	40.9	2.8–169.3	32.4	8.0–153.0	
HGF	221.1	127.8–458.6	277.2	93.4–589.1	
Leptin	40,208.9	12,646.1–12,7802.3	50,185.7	10,869.2–240,758.2	0.0451
VEGF-C	911.7	350.6–3110.3	881.6	169.4–2508.0	
VEGF-D	151.9	3.7–481.9	150.1	27.4–469.8	
EGF	21.8 ^d^	1.5–119.1	21.8 ^d^	1.1–70.4	
Endothelin-1 ^b^	-	-	-	-	
FGF-1 ^b^	-	-	-	-	
FGF-2	120.4	65.8–240.7	120.4	65.8–240.7	
IL-8	4.2	0.6–21.2	4.4	1.7–14.3	
PLGF	8.6	1.8–36.9	7.1	1.8–265.8	
VEGF-A	67.4	6.8–771.8	77.9	6.8–225.2	

^a^*p* values were calculated using the non-parametric Mann–Whitney test; values below 0.05 were considered statistically significant; ^b^ below the detection limit; ^c^ EC patients: n = 38, control patients: n = 37, one measurement was below the detection limit; ^d^ EC patients: n = 30, control patients: n = 34, other measurements were below the detection limit.

## Data Availability

The data presented in this study are available on request from the corresponding author. The data are not publicly available due to data privacy restrictions.

## Supplementary Materials

The following are available online at https://www.mdpi.com/2077-0383/10/4/765/s1, Table S1: Clinicopathological characteristics of the patients with endometrial cancer, Table S2: Patient medication intake within 7 days prior to enrolment, Table S3: Assay Sensitivities (minimum detectable concentrations, pg/mL).
