# Supplementary material for: Tie-2, G-CSF, and Leptin as Promising Diagnostic Biomarkers for Endometrial Cancer: A Pilot Study
Source: J Clin Med. 2021 Feb 15;10(4):765. doi: 10.3390/jcm10040765 (PMC7918088; doi:10.3390/jcm10040765)
Supplement: Supplementary file 1 [file jcm-10-00765-s001.pdf]

Supplementary

Supplementary Table 1. Clinicopathological characteristics of the patients with endometrial cancer.

Characteristic	Subgroup	Cases (n = 38) [n (%)]
Histopathologic subtypes	Well differentiated endometrioid carcinoma (G1)	21 (55.2)
	Moderate differentiated endometrioid carcinoma (G2)	11 (28.9)
	Poorly differentiated endometrioid carcinoma (G3)	6 (15.8)
Presence of metastases	No	31 (81.6)
	Yes	7 (18.4)
Depth of myometrial invasion	No invasion	11 (28.9)
	<50% of the myometrium	15 (39.5)
	>50% of the myometrium	12 (31.6)
Lymphovascular invasion	No	33 (86.8)
	Yes	5 (13.2)
FIGO staging	IA	23 (60.5)
	IB	9 (23.7)
	IIIA	2 (5.3)
	IIIB	1 (2.6)
	IIIIC	3 (7.9)

FIGO: International Federation of Gynecology and Obstetric.

Supplementary Table 2. Patient medication intake within 7 days prior to enrolment. Medications are categorized according to the Anatomical Therapeutic Chemical (ATC) classification system, and the corresponding active ingredient of the drug is listed. Number of control patients and patients with EC taking medications is reported for each represented ATC group.

ATC code	Medication classification	Active ingredients	Number of pts. using medication	
			Controls	Cases
A02	Acid-related disorders	Pantoprazole, esomeprazole, omeprazole	2	2
A10	Used in diabetes	Metformin, glipizide, insulin aspart, insulin detemir, gliclazide, repaglinide	5	4
A11	Vitamins	Alfacalcidiol, cholecalciferol	2	0
B01	Antithrombotic	Acetylsalicilic acid, warfarin	3	3
B03	Anti-anemic	Ferric oxide polymaltose complexes	1	0
C02	Antihypertensives	Moxonidine, doxazosin	1	1
C03	Diuretics	Indapamide, chlortalidone	6	3
C07	Beta blockers	Bisoprolol, carvedilol, metoprolol, propranolol, nebivolol	13	7
C08	Calcium channel blockers	Amlodipine, lacidipine, lercanidipine, nifedipine, diltiazem	6	5
C09	Acting on renin-angiotensin system	Perindopril, enalapril, irbesartan, valsartan, ramipril, telmisartan, zofenopril, losartan, candesartan, trandolapril	23	21
C10	Lipid-modifying agents	Atorvastatin, rosuvastatin, fenofibrate, simvastatin, fluvastatin	10	9
G04	Urologicals	Darifenacin	1	0
H03	Thyroid therapy	Levothyroxine	3	4
J01	Antibacterial for systemic use	Ciprofloxacin	1	0
L02	Endocrine therapy	Tamoxifen	2	0
M01	Anti-inflammatory, antirheumatic	Diclofenac, naproxen, meloxicam	2	1
M04	Anti-gout	Allopurinol	0	1
M05	For treatment of bone diseases	Ibandronic acid, risedronic acid	1	0
N02	Analgesics	Acetylsalicylic acid, paracetamol, propyphenazone, eletriptan	3	0
N03	Anti-epileptics	Valproic acid	0	1
N05	Phycholeptics	Olanzapine, risperidone, zolpidem, quetiapine	1	3
N06	Psychoanaleptics	Fluoxetine, citalopram, escitalopram	0	1
R03	For obstructive airway diseases	Fluticasone, montelukast, budesonide, tiotropium, ipratropium, fenoterol, salbutamol	0	2
R06	Antihistamines for systemic use	Fexofenadine	1	0

Supplementary Table 3. Assay Sensitivities (minimum detectable concentrations, pg/mL).

Minimum Detectable Concentration (MinDC) is calculated using MILLIPLEX® Analyst 5.1. It measures the true limits of detection for an assay by mathematically determining what the empirical MinDC would be if an infinite number of standard concentrations were run for the assay under the same conditions. Information is provided for Human Angiogenesis Magnetic Bead Panels 1 and 2 Cat# HAGP1MAG-12K and HANG2MAG-12K.

Analyte	MinDC pg/mL	MinDC+2SD pg/mL
Angiopoietin-2	3.0	3.0
BMP-9	1.2	1.4
EGF	1.0	1.2
Endoglin	17.0	22.6
Endothelin-1	1.4	1.9
FGF-1	4.6	7.4
FGF-2	8.6	10.5
Follistatin	11.1	13.6
G-CSF	5.4	6.4
HB-EGF	0.4	0.6
HGF	8.5	10.4
IL-8	0.2	0.3
Leptin	42.8	51.9
PLGF	0.7	0.9
VEGF-A	8.1	10.6
VEGF-C	7.6	10.7
VEGF-D	1.9	2.4

Analyte	Overnight Protocol (n = 9 Assays)	
	MinDC (pg/mL)	MinDC+2SD (pg/mL)
Angiostatin	348.3	1081.9
sAXL	2.7	5.6
sc-Kit/SCFR	10.5	30.5
sHer2	5.0	11.9
sHer3	6.1	17.9
sE-Selectin	116.6	247.8
sHGFR/c-Met	10.4	24.2
Tenascin C	6.2	20.2
PDGF-AB/BB	6.4	24.2
sIL-6Rα	7.4	15.1
sTie-2	12.2	35.9
Thrombospondin-2	23.5	118.6
sNeuropilin-1	54.9	151.0
sEGFR	16.0	37.9
suPAR	36.9	129.5
sVEGFR1	2.1	5.1
sVEGFR3	82.9	233.0
sPECAM-1	6.3	15.5
Osteopontin	29.6	73.9
sVEGFR2	19.2	42.9
